# Controlled Human Malaria Infection Studies in Africa—Past, Present, and Future

**DOI:** 10.1007/82_2022_256

**Published:** 2024-01-01

**Authors:** Elizabeth Kibwana, Melissa Kapulu, Philip Bejon

**Affiliations:** Bioscience Department, KEMRI-Wellcome Trust Research Program, Kilifi, Kenya; KEMRI-Wellcome Trust Research Program, Kilifi, Kenya

## Abstract

Controlled human infection studies have contributed significantly to the understanding of pathogeneses and treatment of infectious diseases. In malaria, deliberately infecting humans with malaria parasites was used as a treatment for neurosyphilis in the early 1920s. More recently, controlled human malaria infection (CHMI) has become a valuable, cost-effective tool to fast-track the development and evaluation of new anti-malarial drugs and/or vaccines. CHMI studies have also been used to define host/parasite interactions and immunological correlates of protection. CHMI involves infecting a small number of healthy volunteers with malaria parasites, monitoring their parasitemia and providing anti-malarial treatment when a set threshold is reached. In this review we discuss the introduction, development, and challenges of modern-day *Plasmodium falciparum* CHMI studies conducted in Africa, and the impact of naturally acquired immunity on infectivity and vaccine efficacy. CHMIs have shown to be an invaluable tool particularly in accelerating malaria vaccine research. Although there are limitations of CHMI studies for estimating public health impacts and for regulatory purposes, their strength lies in proof-of-concept efficacy data at an early stage of development, providing a faster way to select vaccines for further development and providing valuable insights in understanding the mechanisms of immunity to malarial infection.

## 1 Introduction

Although there has been substantial progress in reducing malaria morbidity and mortality in the last 15 years, there were still an estimated 241 million cases of malaria in 85 malaria endemic countries in 2020, with 95% of cases occurring in sub-Saharan Africa, and progress has stalled in recent years. Children under five years bear the brunt of the burden accounting for 77% of all malaria deaths (WHO. World Malaria report 2021). Increased funding in malaria control measures, such as indoor residual spraying (IRS), insecticide-treated bed nets (ITNs), intermittent preventive treatment (IPT) in pregnancy, and treatment with artemisinin-based combination therapy (ACT), have contributed to the decline in malaria cases (Casper et al. 2017). However, the number of drug resistance and insecticide-resistant cases are also on the rise (WHO 2018, 2010), threatening the WHO’s vision of a world free of malaria. Vaccines are one of the most cost-effective public health interventions and have been instrumental in the elimination campaigns of other infectious diseases such as smallpox (Henderson 1987) and polio (John 2009). An effective malaria vaccine combined with the various control strategies will undoubtedly contribute to the elimination of malaria.

Controlled human malaria infection models (CHMI) are considered a powerful tool to assess the efficacy of anti-malarial drugs and vaccines as well as help in understanding and defining the precise mechanism by which immune cells contribute to controlling malaria infection (host/parasite interactions). CHMI entails deliberately infecting volunteers with the malaria parasite, the study endpoint is the detection of a pre-determined density of blood-stage parasites, at which point a study subject is treated with a curative regimen of an anti-malarial drug. The monitoring of infection is carried out by microscopy and/or more recently a sensitive quantitative polymerase chain reaction (qPCR) based on 18S ribosomal RNA gene transcripts. The sensitivity of qPCR can be as low as 20 parasites/mL of blood, compared to light microscopy with a sensitivity of 5000 parasites/mL (Bejon et al. 2006). The main strength of CHMI models lies in their defined timing and inoculum dose, which allows associations to be drawn between exposure, immune responses, and ultimately protection, both at the individual level and between experimental groups (Yap et al. 2020).

Historically, deliberately infecting humans with malaria has been used since the 1920s for the treatment of neurosyphilis (Austin et al. 1992; Collins and Jeffery 1999), for which the psychiatrist Julius Wagner-Jauregg was awarded the Nobel Prize in Medicine. In 1986 investigators at Walter Reed Army Institute of Research (WRAIR) conducted the first well-documented human malaria challenge study in the modern era. Six malaria naïve volunteers were infected via the bites of laboratory-reared female Anopheles mosquitoes infected by feeding on *P. falciparum* cultures (Chulay et al. 1986). Since then CHMI has been used safely and successfully in thousands of volunteers worldwide (Table 1) and is routinely carried out in the US Military Malaria Vaccine Program (Spring et al. 2014); the University of Maryland, USA (Friedman-Klabanoff et al. 2019); Radboud University Nijmegen Medical Centre (RUNMC), the Netherlands (Sauerwein et al. 2011); and the University of Oxford, UK (Duncan and Draper 2012). The majority of CHMI studies have been carried out in naïve populations, the major benefit of conducting CHMI studies in naïve populations is it indicates what immune parameters to look out for in exposed individuals.

This review will focus on the establishment of *P. falciparum* sporozoite CHMI models in Africa, as well as the progress, current, and recent advances, and challenges of using this model in malaria exposed individuals.

## 2 Conduct of CHMI

**Fig. 1:**
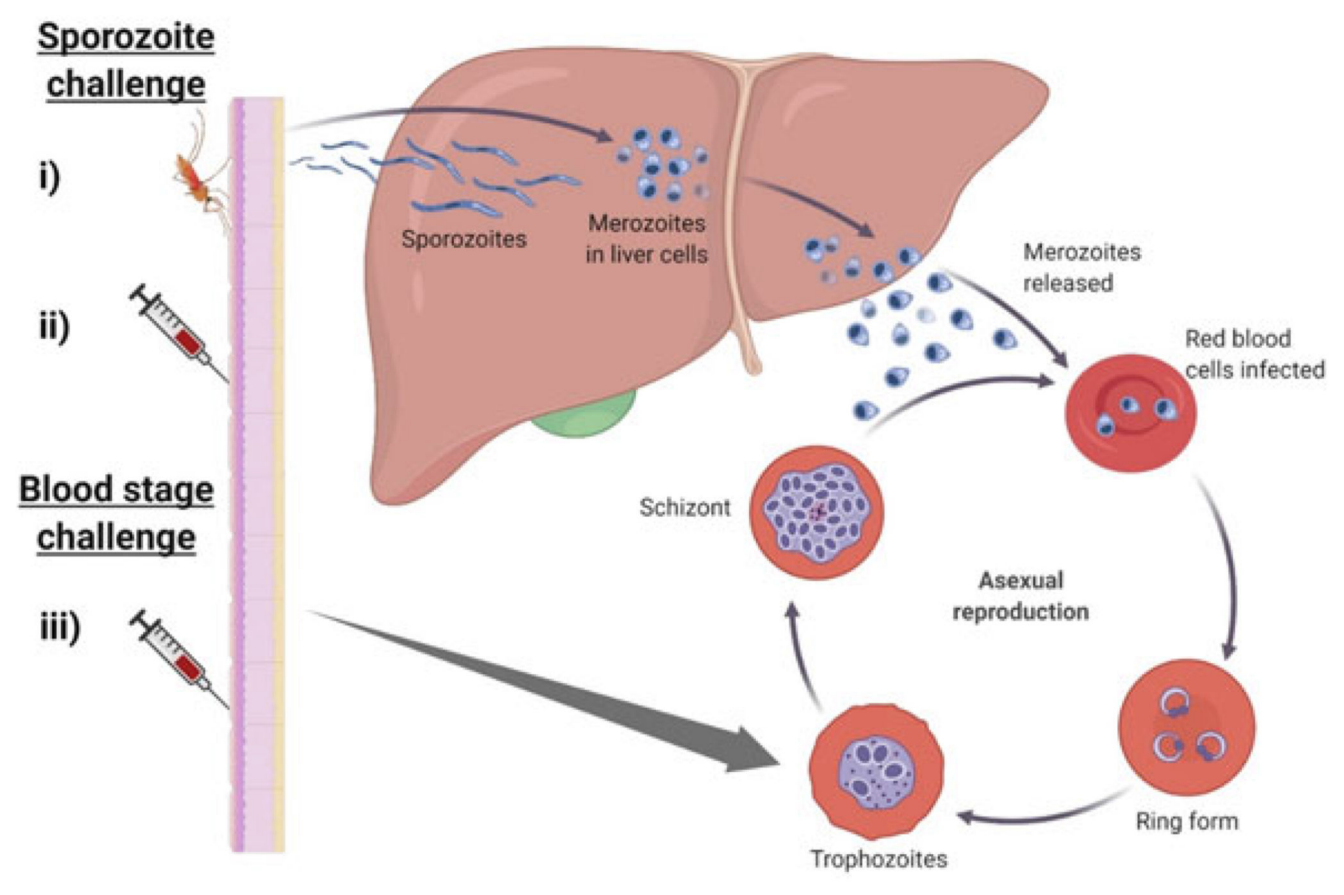
Controlled human malaria infection stages. *P. falciparum* life cycle begins when infected sporozoites are injected into the skin. The sporozoites are rapidly transported via the bloodstream to the hepatocytes in the liver. Within the hepatocytes, the parasite matures, differentiates, and undergoes several cycles of asexual multiplication releasing merozoites into the bloodstream. Once released the merozoites quickly infect erythrocytes and undergo synchronous cycles of asexual replication, progressing through the ring stage, trophozoite stage before finally into the schizont stage. Administration of challenge can be either through sporozoites **(i)** mosquito bite through the skin or **(ii)** needle and syringe via intradermal (ID), direct venous injection (DVI), or intramuscular (IM). Sporozoites travel to the liver where they replicate and are released into the blood. Alternatively **(iii)**, infected red blood cells can be administered directly into the blood, bypassing the liver beginning blood stage replication

The *Plasmodium* life cycle involves two hosts: mosquitoes and humans. Within the human host, the cycle is broadly divided into three stages: (i) pre-erythrocytic stage (liver stage) (ii) erythrocytic stage (blood-stage), and (iii) sexual stage; all represent potential vaccine targets (Duffy and Patrick Gorres 2000). There are two main ways that CHMI can be initiated (Fig. 1): **the sporozoite challenge**; this can be carried out by the traditional inoculation of sporozoites by lab-reared mosquito bites (Epstein et al. 2007; Verhage et al. 2005) or by the direct inoculation via needle and syringe of a known amount of sporozoites (Sheehy et al. 2013; Billingsley 2014; Mordmüller 2015) and ***Plasmodium*-infected red blood cells**, so-called induced blood-stage malaria (IBSM) (Duncan and Draper 2012).

### 2.1 Sporozoite Challenge (Mosquito-Bite and Injection Challenge Studies)

Direct inoculation with sporozoites allows for the development of both liver stage and blood-stage infection and is particularly useful for studying vaccine candidates that target the sporozoite/liver stages. Although mosquito bite challenge would be the most preferred method as it mimics natural infection it does have its limitations, the challenge dose is variable as it is not possible to control the number of sporozoites being injected by the mosquito plus the method was logistically difficult to reproduce and is restricted to only a few research centers with insectary facilities and trained staff (Epstein 2013).

Sanaria Inc., a biotechnology company in Rockville, Maryland, spent the last 10 years addressing the challenges of producing and purifying sporozoites and have developed a method that produces aseptic, purified, cryopreserved, infectious NF54 *P. falciparum* sporozoites (PfSPZ) for injection that complies with regulatory standards. This offers a standardized and reproducible method of conducting CHMI studies (Billingsley 2014; Roestenberg et al. 2009a, 2013; Vanderberg 2007) because of this CHMI by injectable PfSPZ is replacing the traditional method of mosquito bite challenge.

As of August 2021, more than 30 clinical trials of PfSPZ based products have been conducted in 6 sites in the U.S., in 4 European and 7 African countries with over 1200 volunteers receiving PfSPZ (NF54) challenge (Sanaria. 2012). These studies are safe, reproducible, and effective in causing infections in all volunteers (Spring et al. 2014; Epstein 2013; Jongo et al. 2019a, 2018; Draper et al. 2018; Goh et al. 2019; Steinhardt 2019; Stanisic et al. 2018). PfSPZ challenges have been administered by intradermal (ID), direct venous injection (DVI), or intramuscular (IM) all routes have shown to be safe, well-tolerated, and infectious, with DVI being the most efficient route (Roestenberg et al. 2013; Steinhardt 2019; Shekalaghe et al. 2014; Bastiaens et al. 2016; Lyke et al. 2015; Gómez-Pérez et al. 2015). Gómez-Pérez and colleagues demonstrated that IM injection of 75,000 PfSPZ and DVI injection of 3200 PfSPZ resulted in infection rates and pre patent periods comparable to the bite of five PfSPZ infected mosquitoes (Gómez-Pérez et al. 2015). Overall, the PfSPZ challenge studies have been reported to be safe and infectious in both malaria naïve and semi-immune malaria populations.

### 2.2 Blood-Stage Challenge (Induced Blood-Stage Malaria (IBSM) Infection)

Although not as frequently used as the sporozoite mode of challenge, inoculation using blood-stage parasites can be done and was often used in the treatment of neurosyphilis patients in the early 1920s. IBSM results in blood-stage infection and is used to investigate blood stage as well as transmission-blocking vaccine candidates.

The first modern blood-stage CHMI was developed by Cheng and colleagues at the Queensland Institute for Medical Research and uses a cryopreserved stock of erythrocytes from parasitemic donors (Cheng et al. 1997). Two malaria naïve volunteers were infected with *P. falciparum* 3D7 parasites via mosquito bite challenge. 13–14 days later, when volunteers were microscopy positive and experiencing fever, 500 ml of blood was collected from each volunteer and cryopreserved. This master cell bank has been used as an inoculum in several IBSM challenge experiments investigating vaccine and drug efficacy (Cheng et al. 1997; Pombo et al. 2002; Duncan 2011; Lawrence et al. 2000). Post blood-stage challenge, monitoring of parasitemia is carried out by microscopy or highly sensitive qPCR assays, volunteers are then treated at a pre-defined parasite density (Cheng et al. 1997; Pombo et al. 2002).

The majority of challenge studies (sporozoites and blood-stage) are performed using *P. falciparum* as it is the most prevalent and pathogenic parasite (WHO. World Malaria report 2020). However, *Plasmodium vivax* has increasingly been recognized as a public health threat particularly in Asia and Latin America (Battle et al. 2019), as such challenge with *P. vivax* has also been achieved (Table 2) in both sporozoite and blood-stage models (McCarthy et al. 2013; Payne et al. 2017; Griffin 2016; Herrera et al. 2009; Arévalo-Herrera 2016). Currently, no modern-day blood-stage challenges have been carried out in Africa.

One of the main advantages of using the IBSM model is the ability to control the inoculum size; unlike the sporozoite challenge where the number of merozoites released from the liver varies as approximately 30,000 merozoites can be released into the bloodstream when a single infected hepatocyte ruptures (Bejon et al. 2005), the starting inoculum in a blood-stage challenge is much lower and tightly controlled which allows a longer period of observation of parasite growth increasing the number of time points at which to collect parasite data. This allows for the ability to calculate accurate parasite multiplication rates (PMR), the PMR is the per cycle fold-change in parasite numbers and is a measure of vaccine efficacy (VE) (Duncan and Draper 2012; Bejon et al. 2005). IBSM model is particularly useful in assessing VE of candidate blood-stage vaccines before introducing them to field studies. Blood stage vaccines are attractive as this is the stage that causes clinical symptoms (Miller et al. 2002). An effective blood-stage vaccine should demonstrate a measurable effect on PMR, especially during homologous challenges (Duncan 2011; Payne et al. 2016).

Recently, Minassian investigated the safety and efficacy of the vaccine candidate *P. falciparum* reticulocyte-binding protein homolog 5 (RH5) using blood-stage CHMI. Naïve volunteers were immunized with 3 doses of RH5 formulated in GlaxoSmithKline’s (GSK) adjuvant system AS01B followed by a blood-stage challenge with 1000 infected erythrocytes. The authors observed a reduction in parasite growth in the blood as well as immune mechanisms that could predict how well the vaccine performed (Minassian et al. 2021).

Blood stage challenge does have its limitations. Firstly, it circumvents the liver stage of parasite development bypassing immune responses induced by vaccination or natural infection at this stage (Sauerwein et al. 2011). This may underestimate VE as some vaccine candidates contain antigens that are shared between both liver and blood stages (Nahrendorf et al. 2015). The lack of liver stage development however, does have an advantage to the participants undergoing *P. vivax* challenge as there is no risk of hypnozoite formation or relapse (Baird et al. 2007; Auburn et al. 2021). Nonetheless, blood-stage challenges may be able to answer some of the questions that the sporozoite challenge cannot address, such as the immune mechanisms that confer protection. Secondly, there is considerable genetic heterogeneity of *P. falciparum* parasites circulating in the field. The ability to test different vaccine candidates against different strains is in both blood-stage and sporozoite challenge is crucial to vaccine development to determine candidates that offer strain transcending immunity (Cooper et al. 2019). To overcome this, several groups are working on expanding parasites strains for use in challenge studies (Stanisic et al. 2015).

Despite the challenges, the effort to eradicate malaria requires an understanding of all parasite stages and it has been demonstrated that NAI is largely against the blood-stage infection (Cohen et al. 1961) and clinical symptoms are observed during this stage. Therefore, blood-stage challenge still plays an important role in complementing the sporozoite challenge model when assessing potential vaccine candidates as well as identifying immune mechanisms of protection.

#### *Plasmodium falciparum* strains used in CHMI studies

In the field *P. falciparum* displays a wide genetic diversity, which is currently not represented by the available laboratory strains for CHMI. There are currently a limited number of defined *P. falciparum* strains used in CHMI: NF54 (an isolate of West African origin); 3D7 (a clone of NF54); 7G8 (a cloned line of the Brazilian IMTM22 isolate) (Burkot et al. 1984); NF135.C10 (a clone derived from a Cambodian isolate) (Teirlinck et al. 2013); and HMP02 (an isolate from Ghana), with the latter available only for a blood-stage challenge (Sheehy et al. 2013; Stanisic et al. 2018; Ponnudurai et al. 1981). When using NF54 or 3D7, the CHMI is considered homologous, since the parasite is identical to the challenge strain. When other strains are used, the CHMI is considered heterologous, and a potentially better predictor of efficacy under conditions of natural transmission, where mosquitoes harbor heterogeneous populations of *P. falciparum* (Richie et al. 2015). Whether these strains are representative of the antigenic diversity found in malaria endemic regions is unclear. The availability of aseptic, purified cryopreserved *Pf* sporozoites from Sanaria make NF54 the most widely used strain in CHMI studies. Moderate cross reactive protection against heterologous challenge and natural exposure has been observed in both naïve adults and semi-immune individuals suggesting that protection is partially strain specific (Schats et al. 2015; Walk et al. 2017; Sissoko et al. 2017; Kapulu et al. 2020; Lyke et al. 2021; Moser et al. 2020; Ishizuka et al. 2016).

Interpretation of data from CHMI studies and in turn vaccine efficacy trials involving challenge need to take into account whether the challenge was homologous or heterologous and whether the strains used are representative of the parasite’s antigenic diversity in that region (Stanisic et al. 2018).

## 3 Establishment of CHMI in Africa

One of the first human challenge studies in Africa was carried out by Alison and colleagues in 1954, where they investigated the protective effect of sickle cell trait against malaria infection. They looked at two groups of adult males; 15 with sickle cell trait and 15 without, the researchers inoculated the volunteers with either blood containing a large number of trophozoites or by biting with heavily infected *Anopheles gambiae*. They demonstrated an infection with *P. falciparum* was established in 14 out of 15 without the sickle-cell trait, compared to only 2 out of 15 in the sickle cell group demonstrating the protective nature of sickle cell trait in malaria (Allison 1954). Another study conducted in Liberia inoculated semi-immune adults with *P. falciparum* via infected *A. gambiae* mosquitoes (Bray et al. 1962). However, the majority of the ‘modern’ CHMI studies have since been conducted in malaria-naïve populations (Stanisic et al. 2018) which eliminates the potential confounder of prior exposure.

Conducting CHMI studies in malaria-endemic areas offers a huge advantage over malaria naïve individuals, firstly it provides an opportunity to understand the effect of naturally acquired immunity and subsequently, the effect it has on a vaccine and/or drug efficacy as well as assessing vaccine safety and efficacy. Secondly, the trial population matches the actual target population having the same genetic background, and lastly, it offers the opportunity to study the effects of co-infections which can influence vaccine and immune responses (Nouatin et al. 2021). To date, *P. falciparum* CHMI studies have been conducted in 6 African countries summarized in Tables 1 and 2.

### Modern day CHMI studies in Africa

The first modern CHMI study in Africa using cryopreserved PfSPZ was in 2012 (Shekalaghe et al. 2014). The study was conducted in 30 healthy adult males living in Dar es Salaam, Tanzania. The volunteers in this study were considered semi-immune as they had not had a clinical episode of malaria for five years, however, had a positive *P. falciparum* lysate serology before CHMI, indicating a previous exposure. Volunteers were divided into two dose groups with one group receiving 10,000 PfSPZ ID and the other 25,000 PfSPZ ID. The study demonstrated PfSPZ challenge was safe, well-tolerated, and infectious in adults with a previous history of malaria exposure with 21 out of 24 volunteers being parasite positive by day 21. Malaria diagnosis was carried out by thick blood smear with qPCR performed retrospectively. Further safety and infectivity studies have been carried out using Sanaria PfSPZ in adults with varying degrees of malaria exposure in Kenya (Kapulu et al. 2020, 2018; Hodgson et al. 2014), Gabon (Lell et al. 2018; Dejon-Agobe et al. 2019), Gambia (Achan 2019), Equatorial Guinea (Jongo et al. 2021) and all studies have demonstrated CHMI to be safe and infectious in these populations despite the route of administration (summarized in Table 3). Administration of sporozoites was done by ID in Tanzanian trial; IM in the Kenyan study and DVI in the Gabon, Gambia, and Equatorial Guinea studies (Table 4).

### CHMI studies to understand naturally acquired immunity and infectivity

Although slow to develop, naturally acquired immunity (NAI) to malaria does exist. Adults who have grown up in an endemic area and are exposed to repeated *P. falciparum* infections develop immunity, first to severe malaria and then to clinical disease (Doolan et al. 2009). NAI is directed mainly against the asexual parasite cycle in the blood and is key to the rational development and deployment of vaccines. NAI to malaria involves both antibody-mediated and cell-mediated immunity and can be divided broadly into 3 stages (i) pre-erythrocytic (ii) erythrocytic and (iii) anti-gametocyte immunity.

Besides being a powerful tool to study vaccine and drug efficacy, CHMI models offer a way to understand the immune mechanism involved in naturally acquired protective immunity and offer a perfect opportunity to study the effect and the mechanisms of NAI on parasite growth kinetics in a well-controlled setting. In addition, challenge models may aid in the possible identification of immunological correlates of protection. Identifying and establishing correlates of protection and/or immunological substitute endpoints can accelerate vaccine development and contribute to new vaccine approaches and strategies (Stanisic and McCall 2021). Studies have been carried out looking at malaria specific immune responses provoked by parasite exposure during CHMI in naïve individuals recently reviewed by Yap and colleagues. They demonstrated CHMI volunteers are either fast/inflammatory responders or slow/antibody mediated responders (Yap et al. 2020).

When using CHMI models in endemic areas, assessment of vaccine or drug efficacy may be complicated by NAI, although arguably given the existence of NAI in the target populations this is an equally important assessment. Asymptomatic infections present prior to CHMI may also complicate assessments (Cooper et al. 2019), although this can be mitigated by screening for infection and using anti-malarial drugs with short half-life before conducting challenge. Studies looking at the effect of natural immunity using the CHMI model in endemic areas are summarized in Table 5.

Overall NAI in semi-immune adults has shown to either: (a) delay the time to diagnosis by qPCR compared to naïve adults or (b) result in low levels of parasitemia detectable by qPCR, but which does not result in meeting thresholds for treatment or (c) results in apparent sterile protection as most recently demonstrated by Kapulu and colleagues (Kapulu et al. 2020).

In regard to naturally acquired immune responses, pre-CHMI, semi-immune adults as expected have higher antibody levels to a wide range of blood- and pre-erythrocytic stage antigens (MSP-1, MSP-3, AMA-1, PfEMP1, GLURP, CSP, EXP-1, LSA-1, whole sporozoite lysate, whole pRBC lysate), and these increase significantly after CHMI when compared to naïve individuals. Possibly explained by natural pre-existing immunity to these antigens which leads to a stronger memory B cell recall response following CHMI. There was however no consistent association between antibody levels and parasite multiplication rate or parasite density across the studies, neither was there a clear association to any functional assays. Interestingly, in all the studies the researchers were able to group the participants into “high/definite exposure” and “ low/minimum exposure”. These groups were determined by previous exposure serology data measurements to pre-defined antigens. In the “high exposure groups”, antibody responses to measured malarial antigens were in most cases higher, time to diagnosis was delayed and they were able to control infection better as shown by lower parasite densities compared to the “low exposure groups”. This may be explained by different exposures quantities and/or exposure to a variety of parasites strains (Obiero et al. 2015). Remarkably, in each study, there was at least one individual who had sterile immunity post-CHMI with the Kenyan cohort noting 33 individuals portraying sterile protection and displaying cross-reactivity protection to the West African strain (NF54) (Kapulu et al. 2020). The mechanism for this is still unknown however more studies are being carried out with larger sample sizes that could explain sterile protection.

A potential complication when trying to understand the effect of NAI using the CHMI model is that the natural *P. falciparum* strains that semi-immune individuals are exposed to are all unknown. The CHMI strain used in the studies done to date is *P. falciparum* NF54, and this does not represent all the antigenically diverse strains naturally circulating in Africa. While this may complicate assessments of the link between exposure and clinical immunity seen in CHMI, on the other hand it provides an opportunity to assess heterologous protection in the context of CHMI, which is a critical issue in vaccine design.

## 4 CHMI Vaccine Efficacy Studies

Vaccines designed to protect against malaria can be divided into three main groups (i) PE vaccines (ii) blood-stage vaccines (iii) transmission-blocking vaccines. PE vaccines are thought to be ideal as they target the first step of infection, which has relatively low parasites numbers and a longer duration of asexual multiplication which would allow the immune system more time to fight infection (Hill 2006; Abuga et al. 2020). PE vaccines aim to induce antibodies that can neutralize sporozoites in the skin and circulation, block hepatocyte invasion by sporozoites, and/or cell-mediated immune responses that target infected hepatocytes (Duffy and Patrick Gorres 2000; Hill 2006; Marques-Da-silva et al. 2020). Vaccines that can prevent PE development would prevent blood-stage infection and hence prevent pathology. Currently, the most clinically advanced malaria vaccine is a pre-erythrocytic vaccine known as RTS, S which has been recommended by WHO for a pilot implementation currently ongoing in three African countries (Adepoju 2019). RTS, S is based on a large segment of the *P. falciparum* CSP, which is present on the surface of sporozoites. A major challenge for malaria vaccine candidates in both CHMI and field studies is the ability to induce strain-transcending protective efficacy.

The CHMI models are widely used to assess VE, they help define the precise mechanism by which immune cells contribute to controlling malaria infection. The model enables the generation of proof-of-concept efficacy data following a phase I trial, providing a faster way to circumvent the costs and risks involved with phase II trials and in turn accelerating the development of malaria vaccines. The model has also been used to aid in the optimization of immunization regimes (Spring et al. 2014; Stanisic 2018; Roestenberg et al. 2017). Table 6 summarizes studies that have used the challenge model to assess VE.

**Fig. 2:**
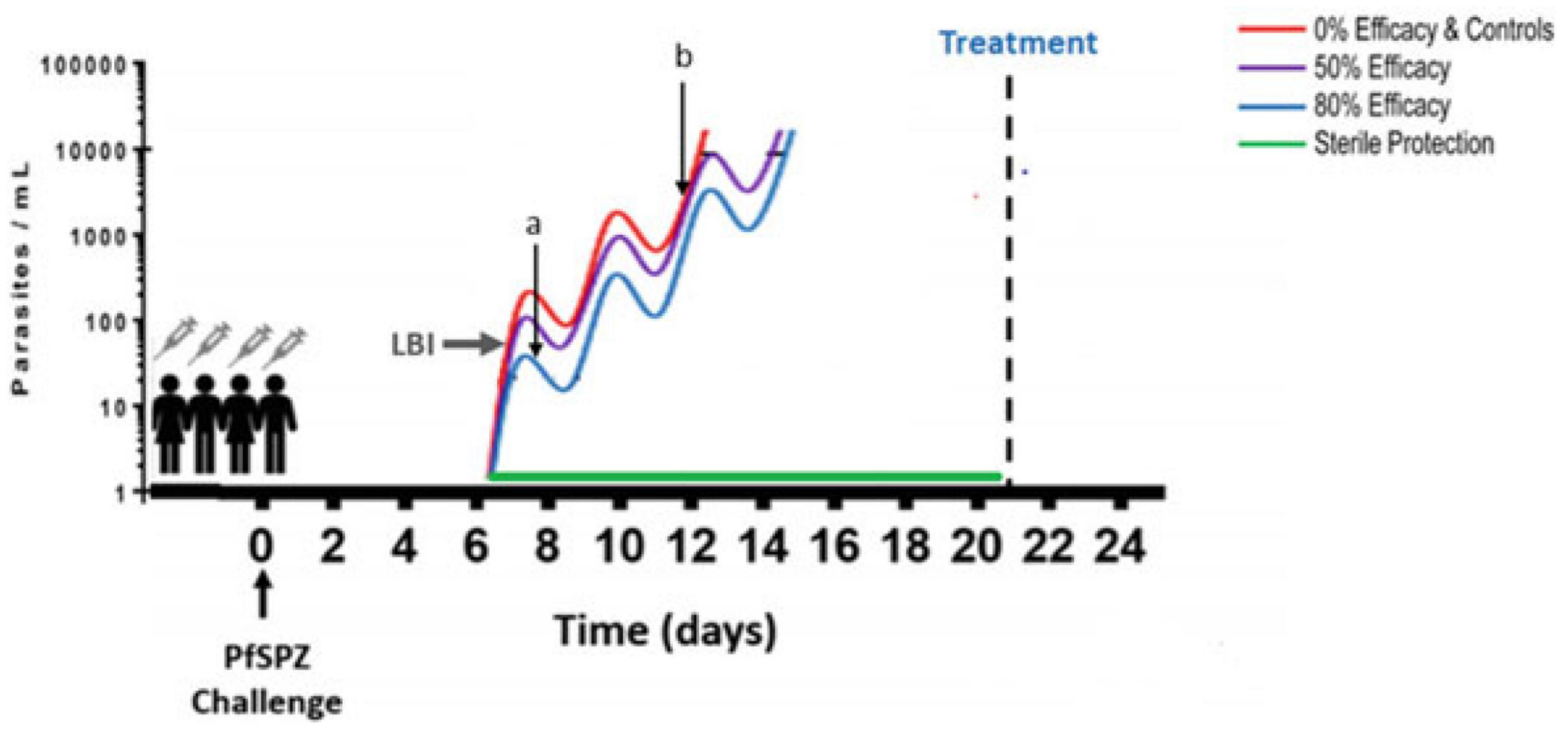
CHMI model for vaccine efficacy (adapted from Draper et al. 2018). The cyclic rise in parasitemia due to asexual reproduction in erythrocytes, monitoring of parasitemia is typically for 21 days before treatment is administered. Following PfSPZ challenge VE is measured by the parasite kinetics and consequently any delay or sterile protection induced by a vaccine. a parasites become detectable at around 20 parasites/ml for qPCR detection **b** and, 2000 parasites/ml for a thick blood smear. LBI, liver to blood parasite inoculum

In CHMI vaccine studies, the volunteer receives a dose/or several doses of the experimental vaccine before being infected with a known number of sporozoites. Following vaccination and challenge, efficacy is measured by analysis of liver to blood parasite inoculum (LBI, Fig. 2). For example, pre-erythrocytic VE may produce sterile protection or partial protection reflected by a reduction in LBI in the vaccinated group relative to controls and a delay to parasitemia monitored by microscopy or qPCR (Draper et al. 2018).

For blood-stage vaccine candidates, the primary assessment is the parasite multiplication rate (PMR). This can be derived from the analysis of qPCR-based parasitemia data from an adequate number of time points and is used to detect differences between vaccinees and control subjects (Bejon et al. 2005; Douglas et al. 2013). Several studies have been carried out to evaluate malaria VE by the challenge model, summarized in Table 6 but few have been carried out in endemic regions.

## 5 Malaria Vaccine Evaluation Studies in Africa Using CHMI

### 5.1 Whole Sporozoite Vaccine Candidates

Whole sporozoite vaccines were one of the first malaria vaccine candidates tested, Nussenzweig and colleagues in the late 1960s reported protective immunity in mice immunized with X-irradiated sporozoites of *Plasmodium berghei* (Nussenzweig et al.1969). Since then, tremendous progress has been achieved using the radiation attenuated SPZ approach in human studies (Richie et al. 2015). Sanaria have developed a PfSPZ vaccine, composed of aseptic, purified, metabolically active, radiation attenuated, cryopreserved sporozoites based on the *P. falciparum strain*, NF54. This vaccine is safe and well-tolerated (Epstein et al. 2007; Lyke et al. 2015) and has demonstrated up to 100% VE against homologous CHMI (same *Pf* strain in vaccine and CHMI) (Draper et al. 2018; Seder et al. 2013; Richie et al. 2015) and short term protection of 80% VE against CHMI with heterologous *Pf* parasites (strain different from that of the vaccine). A study carried out by Lyke and colleagues in 2017 showed 5 of 6 naïve adults previously protected from homologous CHMI were protected against CHMI with Pf7G8 clone (Brazilian in origin), demonstrating durable protection against both homologous and heterologous challenge with PfSPZ vaccine (Lyke et al. 2017).

Furthermore, Sanaria’s PfSPZ vaccine has similarly shown promise in malaria endemic areas. In a study carried out in Tanzania, healthy male adult volunteers were immunized with 5 doses of the PfSPZ vaccine at 1.35 × 10^5^ or 2.7 × 10^5^ followed by homologous DVI CHMI, conducted 3 weeks after the final dose, to assess VE (Jongo et al. 2018). In the group receiving the lower dose, VE against homologous CHMI was 5.6% by analysis of proportions (i.e., 1/18 volunteers protected), while in the higher dose group the VE was 20% (4/20 volunteers were protected). At subsequent re-challenge 24 weeks after vaccination, all four protected individuals were uninfected against a second homologous CHMI. This same immunization regimen had been used in a trial in the United States that gave 92 and 65% VE against 3- and 24-week homologous CHMI (Epstein et al. 2017). The researchers also showed that there was a significant delay to parasitemia by qPCR in volunteers who received the higher PfSPZ dose compared to the controls when challenged with homologous CHMI, suggesting a vaccine induced reduction of sporozoites in the liver (Jongo et al. 2018).

An earlier study carried out in Mali using the same dose regimen (5 doses of 2.7 × 10^5^) demonstrated vaccine protection against natural heterologous *Pf* parasites. VE during the 24 weeks after last vaccine dose was 52% by time to infection analysis and 29% by analysis of proportions (Sissoko et al. 2017) [i.e. similar to the Tanzanian cohort result of 20% (Jongo et al. 2018)]. When compared to naïve adults in the United States, the Mali and Tanzanian cohorts had relatively low VE and anti-CSP responses. In the US study, 6/6 of the volunteers were protected against mosquito bite homologous challenge and also had much higher vaccine-induced antibody and T cell responses (Seder et al. 2013; Epstein et al. 2017). The Tanzanians did however have significantly higher antibody responses than the Malians. This difference may be explained by the cumulative history of *P. falciparum* exposure in the Mali and Tanzania cohorts which may lead to a down-regulation of immune responses to the PfSPZ vaccine. The Malian cohort was from a region of intense malaria transmission whereas the Tanzanian cohort came from an area of low transmission. More recently, a study looking at the safety and efficacy of a higher 3 dose (1.8 × 10^6^) PfSPZ regimen in Mali, observed a VE of 51% (by time to infection analysis) against natural heterologous *P. falciparum* infection (Sissoko et al. 2021) similar to the 52% VE (by time to infection analysis) observed in an earlier trial (Sissoko et al. 2017).

To determine whether increasing the dose of PfSPZ would increase VE an age de-escalation study involving adults, followed by adolescents, children, and finally, infants were carried out in Tanzania. VE against homologous CHMI was assessed in adults only. Immunization doses were increased (to 3 doses of 9.0 × 10^5^ *Pf*SPZ or 1.8 × 10^6^ *Pf*SPZ by analysis of proportions). The vaccine was safe and well-tolerated in all age groups, including the infants (Jongo et al. 2019b). In the adult group receiving 9.0 × 10^5^ *Pf*SPZ, VE was 100% however increasing the dose to 1.8 × 10^6^ *Pf*SPZ significantly reduced VE to 33% (Jongo et al. 2019a). Antibody and T cell responses were highest in the higher dose group (1.8 × 10^6^ *Pf*SPZ).

Jongo and colleagues carried out safety, immunogenicity, and VE study against PfSPZ Vaccine and PfSPZ-CVac [infectious PfSPZ Challenge administered to subjects taking chloroquine chemoprophylaxis (Bastiaens et al. 2016)] in Equatorial Guinea (Jongo et al. 2021). Adults received 3 doses of either 2.7 × 10^6^ PfSPZ vaccine or 1.0 × 10^5^ PfSPZ-CVac challenge. VE was assessed by DVI CHMI and calculated based on the first positive qPCR. VE from PfSPZ vaccine was 27%, while VE from PfSPZ-CVac was 55% against homologous CHMI. Similar to the Tanzanian trial where they increased the dose of PfSPZ to 2.7 × 10^6^ (Jongo et al. 2019a), a high PfSPZ vaccine dose (2.7 × 10^6^) also decreases VE in this population. The authors do plan on addressing this through another study immunizing with a lower dose of PfSPZ. There was no difference in solicited adverse events (SAE) between the PfSPZ vaccinees and controls however the PfSPZ-CVac vaccinees had more SAE than their control group probably related to symptoms attributed to parasitemia (Jongo et al. 2021). Within the two groups, there was an association between PfCSP antibodies and protection.

In summary, the PfSPZ vaccine is safe and tolerable in adults and infants living in endemic areas, however, VE is much lower in semi-immune adults when using either homologous and/or heterologous CHMI (Jongo et al. 2018, 2020; Sissoko et al. 2017; Shekalaghe et al. 2014; Healy et al. 2017), and in field studies with heterologous *Pf* parasites (Sissoko et al. 2017) when compared to the studies carried out in naïve adults (Lyke et al. 2017; Mordmüller et al. 2017; Epstein et al. 2017). The studies conducted so far have demonstrated the efficacy of PfSPZ vaccine is dose-dependent and regimen dependent, with VE efficacy improving when either or both are changed in both homologous/heterologous CHMI. Studies carried out in malaria endemic areas have used a much higher immunization dose to try and overcome the low VE, however increasing the dose to a point is detrimental to VE as demonstrated in the Tanzania trial (Jongo et al. 2020).

Another explanation for the lower VE observed in heterologous challenge could be due to the challenge strain not representing the genetic diversity of the parasite in the field. Moser and colleagues conducted whole genome sequencing of the Sanaria PfSPZ vaccine strain NF54, and compared this against the strains used in heterologous challenge (7G8, NF166.C, and NF135.C10) as well as a collection of clinical isolates relative to the reference strain 3D7 (a clone of NF54) (Moser et al. 2020). The authors confirmed that NF54 and 3D7 are genetically similar and, as expected based on their respective geographical origins, that 7G8, NF166.C8, and NF135.C10 were genetically very distinct from NF54 and 3D7 with tens of thousands of variants detected between them. These variations may impact the ability of the immune system primed with NF54 to recognize the other *Pf* strains, impairing VE against heterologous CHMI (Moser et al. 2020). Caution should be taken interpretating outcomes of VE when using challenge models as field VE may be overestimated when measured by homologous CHMI (Sissoko et al. 2021). The same applies when comparing VE results between studies conducted between different study sites, target populations and different follow-up time points. The latter being is particularly important when using time to infection analysis in field studies. Further studies are underway to overcome the low VE and immune responses seen in endemic areas by optimizing doses and varying the timing.

### Blood-stage vaccine candidate

In 2015 Dejon-Agobe and colleagues conducted a phase 1 clinical trial to evaluate the safety, tolerability, immunogenicity, and efficacy of CAF01 and aluminum hydroxide as adjuvants for the malaria vaccine candidate GMZ2 in healthy Gabonese adult volunteers using standardized CHMI to assess efficacy (Dejon-Agobe et al. 2019). GMZ2 is an asexual blood-stage candidate; a fusion protein of fragments of *P. falciparum* glutamate-rich protein (GLURP) and MSP3. 50 semi-immune healthy adults were vaccinated with three doses at 4-week intervals of GMZ2 formulated in either CAF01, alhydrogel, or a control vaccine. CHMI was carried out 13 weeks post-last dose after treating the volunteers to clear any *P. falciparum* infections. Anti-GMZ2, GLURP, and MSP3-specific IgG were detected at baseline with anti-GMZ2 and GLURP significantly increasing in the vaccinated group. Baseline vaccine-specific IgG levels predicted the time to development of malaria. Despite this GMZ2 vaccination did not result in protection in this population. The primary efficacy endpoint was malaria, defined as a positive blood smear and at least 1 malaria-related symptom. This study demonstrated that CHMI is an efficient way to down-select blood-stage vaccine candidates derived from associated studies in individuals living in endemic areas.

## 6 CHMI Model as a Vaccine?

### 6.1 *Pf*SPZ Chemoprophylaxis Vaccination (*Pf*SPZ CVac)

Some research groups are now using a similar inoculation of live parasites as done in the “CHMI model” as an immunization strategy. In 2009, Roestenberg et al. showed protection to homologous challenge in naïve adults can be achieved with the inoculation of live, whole sporozoites. They tested two groups of naïve adults; one group was immunized with mosquitoes that were infected with the NF54 strain of *P. falciparum* once a month for 3 months while receiving a prophylactic regimen of chloroquine. The control group was immunized with un-infected mosquito bites. All subjects received chloroquine prophylaxis. Chloroquine kills parasites in the asexual stages but allows parasite infection of the liver (the pre-erythrocytic stage) (Zhang et al. 1986). Eight weeks after the last immunization both groups were challenged by exposure to the bites of five mosquitoes that were infected with the same homologous NF54 strain. The researchers observed no blood-stage parasites in the vaccinated group compared to the controls, where blood-stage asexual stages were observed in blood smears (Roestenberg et al. 2009b). The Sanaria PfSPZ chemoprophylaxis vaccination (PfSPZ CVac) approach duplicates Roestenberg’s work but uses aseptically, purified, cryopreserved, infectious NF54 *P. falciparum* sporozoites in an injectable regimen with chloroquine being administered as the chemoprophylaxis. The PfSPZ CVac model has shown to be safe and well-tolerated in healthy naïve adults and it prevented infection in 100% of volunteers who underwent CHMI by DVI ten weeks after the last immunization after three doses (Mordmüller et al. 2017). Urbano demonstrated that the PfSPZ CVac model is also safe and well-tolerated in adults living in Equatorial Guinea (Urbano et al. 2018).

## 7 Summary and Conclusion

There has been steady progress over the last four decades in anti-malarial vaccine candidates especially with the development of PfSPZ based products from Sanaria which has allowed research centers in endemic areas to use this model. The named studies above have demonstrated that CHMI can be carried out in endemic settings and are safe and infectious in semi-immune adults. One of the main unanswered questions in malaria immunology is what is the correlate of protection in humans? The CHMI model offers an ideal opportunity to answer this question. It is a versatile tool that can be used to; assess vaccine and drug efficacy; immunization strategy, and to understand host-parasite interactions. It is extremely valuable when assessing immunogenicity and dosage in early phase I clinical trials as it allows the use of small sample sizes to predict the effectiveness of a vaccine in the target population. Although, the sample sizes used in these studies were small and possibly contributed to some of the results obtained such as lack of consistent association in antibody to levels to protection, which has varied from low through intermediate. CHMI has proved to be an informative study design and with the increase in sites and participant numbers in endemic areas some of these major questions such as correlates of protection may yet be answered.

One limitation of CHMI studies is it does not mimic the natural route of infection; although all routes are infectious, the many CHMI studies described here use direct venous injection (DVI) which circumvents the skin completely. The dermis is the first stage of infection and is critical for the establishment of infection as well as induction of the host's immune response (Sinnis and Zavala 2012). Studies using DVI as the inoculation route will have implications when assessing both NAI and VE. Additionally, it has been shown, in mice models that mosquitoes “reset” parasite virulence (Spence et al. 2015) which may also confound VE endpoints. Another limitation is that only four *P. falciparum* strains are available for use in CHMI studies (Teirlinck et al. 2013). *Plasmodium* spp are genetically diverse. For future CHMI studies, a larger variety of well characterized parasite strains are needed to reflect the heterogenicity of the various endemic areas. Heterologous challenges could then be used to demonstrate the efficacy of vaccine candidates against a diverse range of parasite strains to determine whether there is strain transcending protection and optimize vaccine formulation and/or regimen prior to field trials. Thus, avoiding the cost of lengthy clinical trials bringing effective vaccines to market more rapidly. However, this is challenging as not all malaria strains are easily laboratory adapted. For manufacturing process, parasites cultures should (i) consistently produce gametocytes and sporozoites; (ii) be sensitive to commonly available anti-malarials and (iii) be geographically and molecularly distinct from 3D7. As a result, only after extensive efforts on > 70 strains was the parasite clone, NF135.C10 identified (Stanisic et al. 2018; Teirlinck et al. 2013).

A standardized and optimized method for the measurement of LBI will also be important to help explain the mechanism of sterile immunity seen in some semi-immune adults. An additional drawback is that CHMI can only be conducted in healthy adult volunteers, which does not necessarily reflect the target population. The malaria burden in endemic regions lies in children under 5 and in pregnant women. CHMI would not be ethical in either population. Given these limitations, it is unlikely CHMI studies would replace the need for larger full-scale phase 3 clinical field trials. However, CHMI models can help prioritize and accelerate novel vaccine candidates allowing relatively small and inexpensive Phase II efficacy trials to take place before larger field trials as well as providing a valuable tool to study host–pathogen interactions.

## Figures and Tables

**Table 1:** Sites conducting controlled human malaria infection

Africa	The Americas	Australia and Asia	The UK and Europe
^a^ KEMRI-Wellcome Trust Research Program, Kilifi, Kenya	^a,b^ The Walter Reed Army Institute of Research (WRAIR), Bethesda, Maryland	^a,b^ QIMR Berghofer Medical Research Institute (QIMR), Brisbane	^a,b^ CCTVM, University of Oxford, Oxford
^a^ Ifakara Health Institute—Bagamoyo, Tanzania	^a,b^ The Malaria Human Challenge Center (HCC), Seattle		^a,b^ Radboud University Nijmegen Medical Centre (RUNMC), the Netherlands
^a^ Medical Research Center Lambaréné (CERMEL), Gabon	^a, b^ The University of Maryland, Maryland		^a, b^ Institute for Tropical Medicine, Eberhard Karls University of Tübingen, Germany
^a^ Malaria Research and Training Center, Donéguébougou, Mali	^a, b^ Malaria Vaccine and Drug Development Center (MVDC), Cali, Colombia		
^a^ EGMVI, Malabo Equatorial Guinea			
^a^ The Medical Research Council Unit (MRC), The Gambia			

a challenge conducted by needle and syringe

b challenge via mosquito bite *P. vivax* CHMI studies have been conducted in Cali, Columbia; WRAIR, Bethesda, Maryland and QIMR, Brisbane

**Table 2:** Overview of induced blood-stage infection studies

Study type	References
*Plasmodium falciparum* Pilot (Safety and infectivity) Vaccine efficacy	Cheng et al. (1997), Pombo et al. (2002), Sanderson et al. (2008), McCarthy et al. (2011) Duncan (2011), Lawrence et al. (2000), Sanderson et al. (2008), Payne et al. (2016), Minassian et al. (2021)
*Plasmodium vivax* Pilot (Safety and infectivity) Vaccine efficacy	McCarthy et al. (2013), Griffin (2016), Herrera et al. (2009), Collins et al. (2020), Herrera et al. (2011) Arévalo-Herrera (2016), Bennett et al. (2016), Lopez-perez (2016)
*Plasmodium malariae* Pilot (Safety and infectivity)	Woodford et al. (2020)

**Table 3:** Summary of CHMI studies carried out in Africa

Trial site	Study type	No. of volunteers	Plasmodium strain	Route of CHMI administration	Malaria outcome^a^	Pre-patent period (days [min-max])	References
Tanzania	Safety, infectivity and effect of prior malaria exposure to CHMI	30	PfNF54	ID	TBS and qPCR^b^	11–13.5	Shekalaghe et al. (2014), Obiero et al. (2015)
	Safety, immunogenicity, and efficacy of PfSPZ vaccine	67	PfNF54	DVI	TBS and qPCR^b^	7.5–9.7	Jongo et al. (2018)
	Safety and immunogenicity in age de–escalation of PfSPZ vaccine	30	PfNF54	DVI	TBS and qPCR^b^	11.1–12.6	Jongo et al. (2019a), Obiero et al.(2015)
Gabon	Safety and infectivity	25	PfNF54	DVI	TBS	7.9–10.6	Lell et al. (2018)
	Safety, immunogenicity, and efficacy of GMZ2 vaccine	50	PfNF54	DVI	TBS and qPCR	NA	Dejon-Agobe et al. (2019)
Gambia	Safety, infectivity and effect of prior malaria exposure to CHMI	19	PfNF54	DVI	TBS and qPCR^b^	9–11	Achan (2019)
Equatorial Guinea	Tolerability, safety, immunogenicity, and efficacy against CHMI of PfSPZ Vaccine versus PfSPZ-CVac	52	PfNF54	DVI	qPCR	8–18 (Group 1: PfSPZ vaccine) 8–16 (Group 2: PfSPZ-CVac)	Jongo et al. (2021)
	Vaccine efficacy of PfSPZ vaccine and regimen optimization	104	PfNF54	DVI	qPCR	–	NCT03590340
Mali	Safety and efficacy of PfSPZ–CVac (PfSPZ challenge under chemoprophylaxis)	62	PfNF54	DVI	qPCR	–	NCT02996695
	Safety and efficacy of a three–dose regimen of PfSPZ vaccine in adults against homologous controlled human malaria infection (CHMI) and natural *P falciparum* infection	56 (pilot study) 120 (main study)	PfNF54 and natural infection	DVI (Pilot study cohort)	TBS and qPCR^b^	NA	Sissoko et al. (2021)
Kenya	Safety, infectivity, and effect of prior malaria exposure to CHMI	28	PfNF54	IM	qPCR^b^	11.8–16.4	Hodgson et al. (2014)
	Controlled human malaria infection in semi–immune Kenyan adult	142	PfNF54	DVI	qPCR	NA	Kapulu et al. (2020)

a Malaria outcome measured by either thick blood smear and/or qPCR.

b qPCR measured retrospectively

Abbreviations: *DVI* direct venous inoculation; *IM* intramuscular; *ID* intradermal; *qPCR* quantitative PCR; *TBS* thick blood smear; *NA* not available

**Table 4:** Ongoing and upcoming CHMI studies in Africa

Trial site	Study type	Description	References
Kenya	Controlled human malaria infection transmission model—Phase A (CHMI-TransMod)	This is to develop a model to test the efficacy of vaccines and/or drugs designed to block transmission of malaria to mosquitoes and to identify the targets of transmission-blocking immunity to malaria	NCT04280692
	Safety, Immunogenicity, and Efficacy of R21/Matrix-M and ChAd63/MVA-ME-TRAP in the Context of Controlled Human Malaria Infection: A Phase IIb Trial in Kenyan Adults	VE—a study to determine if new types of malaria vaccines are safe, effective, and lead to immunity—Efficacy assessed by CHMI	NCT03947190
Tanzania	Safety, Immunogenicity, and Efficacy of PfSPZ Vaccine in HIV Negative and HIV Positive Tanzanian Adults	Efficacy assessed by CHMI	NCT03420053
Mali	Safety, Immunogenicity, and Protective Efficacy of Radiation Attenuated Plasmodium Falciparum NF54 Sporozoites (PfSPZ Vaccine) in Healthy African Adults in Mali	Efficacy assessed by CHMI	NCT02627456

**Table 5:** Summary of studies looking at the effect of NAI

Country	Exposure history	No. of participants/groups	Route	Dose	Time to parasite positivity (TBS)	Time to parasite positivity (qPCR	No. of participants infected by D2l	Immunological assay carried out	Summary of findings	References
The Netherlands^a^	Naïve	6	ID	2500	13.0	10.6	5/6	ELISA: CSP, LSA-1, AMA-1, EXP-1, and *P.f* lysate ^c^ Cellular responses: IFN-γ	Tanzania cohort—higher *P. falciparum* specific Ab levels (pre/post CHMI); Pre-patency period correlated with pre-CHMI anti-CSP Abs Dutch cohort—Higher IFN-γ post-CHMI by both CD4+ and CD8+ T cells when compared to Tanzanian cohort	Obiero et al. (2015), Roestenberg et al. (2012)
		6		10,000	12.7	10.34	5/6			
		6		25,000	13.0	9.95	5/6			
Tanzania^a^	Semi-immune	12	ID	10,000	15.4	12.6	11/12			
		12		25,000	13.5	11.1	10/11			
United Kingdom^b^	Naïve	6	ID	2500	13.0	NA	5/6	ELISA: Schizont extract^c^, MSP-1, AMA-1, and RH5 Functional in vitro assays: GIA and ADRB	KCS cohort—significant difference in anti-MSP-1/AMA-1 and ADRB activity between the MinExp and DefExp groups pre/post CHMI; Pre-CHMI antibodies to AMA1 and schizont extract correlated with PMR One individual demonstrated sterile immunity (qPCR negative) UK cohort—Antibody levels to MSP-l/AMA-1 were significantly increased post-CHMI; No ADRB activity post-CHMI	Sheehy et al. (2013), Hodgson (2016)
		6	IM	2500	12.7	NA	3/6			
		6	IM	25,000	13.0	NA	6/6			
Kenya^b^	Varying	2 (MinExp ^c^)	IM	25,000	12.5	12.6	2/2			
		2 (DefExp ^c^)		25,000	11.9	16.4	2/2			
		2 (MinExp ^c^)		75,000	11.8	11.8	2/2			
		2 (DefExp ^c^)		75,000	13.25	13.3	2/2			
		10 (MinExp ^c^)		125,000	11.8	12.0	10/10			
		10 (DefExp ^c^)		125,000	12.3	11.9	10/10			
The Gambia	semi-immune	9 (sero-high ^c^)	DVI	3200	14.0	11.0	8/9	ELISA: NF54 sporozoite or schizont extract Functional in vitro assays: GIA, SIA	Antibody responses to sporozoite, schizont extract and GI activity was significantly higher in sero-high group than sero-low One individual demonstrated sterile immunity (qPCR negative)	Achan (2019)
		10 (sero low ^c^)			13.5	9.0	10/10			
Gabon	Naïve & semi-immune	5 NI	DVI	3200	12.6	7.9	5/5	ELISA: PfCSP, PfEXPl, and PfMSPl	NI developed clinical malaria; time to parasitemia: NAI and SCT > NAI; low PMR in LA and IS No association between antibody levels before CHMI and developing parasitemia in IA and IS (TBS) Four individuals achieved sterile immunity	Lell et al. (2018)
		11 IA			16.9	7.9	7/11 (9/11 qPCR)			
		9 IS			19.1	10.6	5/9 (7/7 qPCR)			
Kenya	Varying	15 (High Transmission)	DVI	3200	NA	NA	12/15	Schizont ELISA ^c^	NAI immunity from East African parasites offers cross-reactive protection to a West African parasite 33 individuals demonstrated sterile immunity (qPCR negative)	Kapulu et al. (2020)
		34 (Low Transmission)			NA	NA	32/34			
		112 (Med Transmission)			NA	NA	65/93			

a Authors compared two cohorts naïve Dutch adults and semi-immune Tanzanian adults subjected to identical CHMI protocol

b Authors compared two cohorts naïve UK adults and semi-immune Kenyan adults subjected to identical CHMI protocol

c individuals were grouped according to their level of exposure as determined by defined antigens

Abbreviations: *DVI* Direct venous inoculation; *ID* Inter-dermal; *IM* Inter-muscular; *NA* not available; *ELISA* Enzyme Linked Immunosorbent Assay; *CSP* Circumsporozoite protein; *LSA-1* Liver-stage antigen 1; *AMA-1* Apical membrane protein 1; *EXP-1* Exported protein 1; *IFN-γ* Interferon-gamma; *MSP-1* Merozoite surface protein 1; *RH-5* Reticulocyte-binding protein homolog 5; *GIA* growth inhibition activity; *ADRB* Antibody-dependent respiratory burst activity assay; *SIA* Sporozoite inhibition assay; *KCS* Kenyan challenge study; *PMR* parasite multiplication rate; *NI* non-immunes (malaria naive or minimally exposed without sickle cell trait); *IA* semi-immunes with lifelong malaria exposure and with hemoglobin (HbAA); *IS* semi-immunes with sickle cell trait (HbAS); *NAI* naturally acquired immunity; *SCT* sickle cell trait

**Table 6:** Summary of CHMI studies used in vaccine evaluation worldwide

Category	*Plasmodium* protein	References
Pre-erythrocytic vaccines	Complete sporozoite	Jongo et al. (2018), Walk et al. (2017), Lyke et al. (2021), Ishizuka et al. (2016), Jongo et al. (2021), Lyke et al. (2017), Mordmüller et al. (2017), Jongo et al. (2019b), Shekalaghe et al. (2014), Sulyok (2021), Roestenberg et al. (2009b), Herrington et al. (1990), Clyde et al. (1973), Seder et al. (2013), Hoffman et al. (2002), Epstein et al. (2017)
	Recombinant CSP	Kester et al. (2009), Rampling et al. (2018), Regules et al. (2016), Kester et al. (2007), Kester et al. (2008), Ockenhouse et al. (2015), Moon et al. (2020), Ballou (1987), Stoute et al. (1997), Tamminga et al. (2013)
	ME-TRAP	Rampling et al. (2018), Afolabi et al. (2016), Hodgson et al. (2015), Andrews et al. (2005), Dunachie et al. (2006), Webster et al. (2005), Mcconkey et al. (2003)
	LSA	Cummings et al. (2010)
Multi-stage vaccine	CSP, PfSSP2, LSA1, MSP1, SERA, AMA1 and Pfs25	Ockenhouse et al. (1998)
Blood stage vaccines	AMA-1	Duncan (2011), Tamminga et al. (2013), Spring (2009), Sheehy et al. (2012), Chuang et al. (2013)
	MSP1; MSP2; RESA	Lawrence et al. (2000)
	MSP1	Sheehy et al. (2012), Patarroyo et al. (1988)
	GLURP and MSP3	Dejon-Agobe et al. (2019)
	RH5	Minassian et al. (2021)

Abbreviations: *CSP* circumsporozoite protein, *ME-TRAP* multiple epitope-thrombospondin-related adhesion protein; *LSA-1* liver Stage Antigen-1; *PfSSP2* Plasmodium falciparum sporozoite surface protein 2; *MSP-1* merozoite surface protein-1; SERA serine repeat antigens; AMA-1 apical membrane antigen 1; *RESA* ring-infected erythrocyte surface antigen; *GLURP* glutamate-rich protein (GLURP); *RH-5* Reticulocyte-binding protein homolog
